# Photochemical Responses of *Parmotrema tinctorum* and *Usnea barbata* to Light Variations in Cerrado Landscapes

**DOI:** 10.3390/plants14172802

**Published:** 2025-09-07

**Authors:** Luciana Cristina Vitorino, Márcio Rosa, Bárbara Gonçalves Cruvinel, Matheus Mendonça de Souza Marques, Alex Marcelino Dos Santos, Layara Alexandre Bessa

**Affiliations:** 1 Laboratory of Agricultural Microbiology, Instituto Federal Goiano, Rio Verde Campus, Rodovia Sul Goiana, Km 01, Rio Verde 75901-970, GO, Brazil; matheusmsm12@hotmail.com (M.M.d.S.M.); alexmarcelinobio@gmail.com (A.M.D.S.); 2 Fazenda Fontes do Saber, Rio Verde Campus, Rio Verde University (UniRV), Caixa Postal 104, Rio Verde 75901-970, GO, Brazil; marciorosa@unirv.edu.br; 3 Laboratory of Metabolism and Genetics of Biodiversity, Instituto Federal Goiano, Rio Verde Campus, Rodovia Sul Goiana, Km 01, Rio Verde 75901-970, GO, Brazil; barbaracruvinel.rv@gmail.com (B.G.C.); layara.bessa@ifgoiano.edu.br (L.A.B.)

**Keywords:** Brazilian savannah, energy dissipation, photobionts, photoinhibition, fluorescence, photosynthetically active radiation

## Abstract

Epiphytic lichens are vital to tropical biodiversity, their distribution shaped by light. *Parmotrema tinctorum* and *Usnea barbata*, common in open Cerrado, endure high radiation, necessitating photoprotection. This study tested the hypothesis that the primary photochemistry of *P. tinctorum* and *U. barbata* responds differentially to light conditions across distinct landscapes of the Brazilian Savanna, to the height at which lichens were sampled, and to radiation levels from different components of the visible spectrum. Our results demonstrate that *P. tinctorum* and *U. barbata* possess efficient photoprotective mechanisms, such as energy dissipation as heat, which enable their survival in the dry and highly illuminated landscapes of the Brazilian Savanna. In particular, stressful environments such as Cerrado and Cerrado Ralo exhibited high DI_0_/RC values, leading to lower photochemical performance in lichen thalli. However, *U. barbata* showed greater resilience to light stress than *P. tinctorum*, likely due to the presence of antioxidant metabolites such as usnic acid. Lichens sampled at higher stem positions and exposed to elevated levels of photosynthetically active radiation (PAR) dissipated less energy as heat and exhibited lower photochemical performance, suggesting photosystem II (PSII) damage under these conditions. Conversely, when different components of the visible spectrum were analyzed separately, increasing light intensities reduced DI_0_/RC and enhanced Pi_ABS in the thalli, highlighting photodamage resistance in *P. tinctorum* and *U. barbata*. The ability of both species to adapt to high-light environments, combined with their physiological plasticity, supports their broad distribution in these tropical ecosystems.

## 1. Introduction

Epiphytic lichens are key components of biodiversity in tropical ecosystems. While generalist species tend to occur in open areas, stenoecious lichens (lichens with a narrow environmental tolerance) are restricted to the shaded interiors of forests [1]. Light is a major factor influencing lichen growth, survival, and distribution [2,3]. However, further research is needed to disentangle the specific effects of light intensity on lichen photobiont photosynthesis.

In open vegetation formations, lichens are exposed to high levels of radiation, experiencing repetitive and prolonged desiccation as part of their poikilohydric lifestyle [4]. However, upon rehydration, their metabolism can reactivate within minutes [5,6]. This remarkable resilience allows lichens to tolerate severe abiotic conditions, such as extreme temperatures and intense radiation [7,8,9], despite their well-documented sensitivity to pollution [10]. Thallus desiccation typically coincides with high solar exposure and lichens that colonize open vegetation areas must have efficient photoprotective mechanisms to prevent photodamage to the algal component [11].

Lichen symbiosis relies on an anatomical structure formed by the mycobiont, which can provide protection to the photobiont against excessive water loss, extreme temperatures, and high irradiance [12,13], conditions characteristic of the dry vegetation formations of the savanna. In these environments, the lichens *Parmotrema tinctorum* (Despr. ex Nyl.) Hale, and *Usnea barbata* (L.) Weber ex F.H. Wigg (both Parmeliaceae) are commonly found in open areas with sparse canopy cover, allowing significant light penetration. In dry climates, increased light exposure on lichen thalli exacerbates desiccation, and photosynthesis under severe desiccation can generate reactive oxygen species (ROS) [14,15,16]. Given these challenges, we investigated how light availability in different Cerrado landscapes influences the energy dissipation capacity and photodamage avoidance strategies of the foliose lichen *P. tinctorum* and the fruticose lichen *U. barbata*.

The amount of light in the environment can differentially affect foliose and fruticose lichens through its influence on humidity control [17]. *P. tinctorum* has thalli with broad lobes [18], with a flat surface, which receive direct radiation. This morphology promotes rapid hydration and dehydration, leading to abrupt fluctuations in metabolic activity. In *U. barbata*, however, the thalli exhibit a branched architecture with a cartilaginous central axis [19], which enhances resistance to desiccation. In this case, part of the thallus is not exposed to direct sunlight. In this structure, diffuse branches and filamentous lateral projections (fibrils) partially shade the thallus. We focused our study on these lichens in the Cerrado biome due to its critical ecological importance. The Cerrado is recognized as the most biodiverse savanna on the planet, harboring a vast number of endemic species of both flora and fauna. Furthermore, it functions as Brazil’s “cradle of waters,” giving rise to rivers that feed the main hydrographic basins of both Brazil and South America [20,21]. Consequently, its conservation is vital for maintaining regional and global ecological balance [22].

A study by Gauslaa and Goward [23] identified patterns in the vertical and horizontal distribution of Parmeliaceae on tree stems in temperate forests, likely influenced by light availability at different canopy heights. Based on this, we hypothesize that the primary photochemistry of *P. tinctorum* and *U. barbata* responds differentially to light conditions across distinct landscapes, the height at which lichens are sampled (which determines light exposure), and radiation intensity from different components of the visible spectrum.

Excess radiation can induce ROS formation, leading to damage to the photosynthetic apparatus, particularly PSII [24,25,26], and in severe cases, even lichen mortality. This occurs when absorbed excitation energy cannot be effectively used for electron transport and must instead be dissipated as heat or fluorescence [27,28]. PSII damage, as well as the species’ ability to dissipate excess energy, can be assessed through primary photochemical parameters measured via chlorophyll *a* fluorescence [29].

Chlorolichens are generally regarded as more resistant to photochemical damage than cyanolichens [30]. In tropical regions, lichens are predominantly associated with chlorophyte photobionts of the orders *Trebouxiales* and *Trentepohliales*, with *Trebouxia* representing the most frequently occurring genus [31]. However, stress tolerance and the physiological potential of lichen photobionts adapted to savanna conditions remain poorly understood. This study investigates the physiological responses of *P. tinctorum* and *U. barbata* to varying light intensities characteristic of the Brazilian Cerrado. By incorporating measurements of PSII activity, we aim to enhance understanding of the regulatory mechanisms underlying their photoprotective strategies.

## 2. Results

The models indicated that random effects associated with trunk height and lichen species influenced various chlorophyll *a* fluorescence parameters (ABS/RC, TR_0_/RC, ET_0_/RC, DI_0_/RC, PHI_DO_, PI_ABS_, PHI_PO_, PSI_0_, and PHIE_0_). However, the simplest model, which considered only Landscape + Species, provided the best explanation for all primary photochemistry variables. The second-best model included Landscape + Species + Height (Table 1).

Based on the F-test results, the Landscape + Species model had a significantly greater effect than three other models in explaining ABS/RC. For ET_0_/RC, this model outperformed most of the evaluated models (five in total). Regarding DI_0_/RC and PHI_DO_, the effect of Landscape + Species was superior to that of three other models. For PI_ABS_, it outperformed only one model, which included Landscape + Species + Height + PPFD + PFDUV + PFDG + PFDR + PFDFR + IRR + PAR. In the case of PHI_PO_, this model performed better than two other models, while for PSI_0_, it surpassed four models. Finally, for PHIE_0_, the Landscape + Species model was superior to three other models.

**Figure 1:**
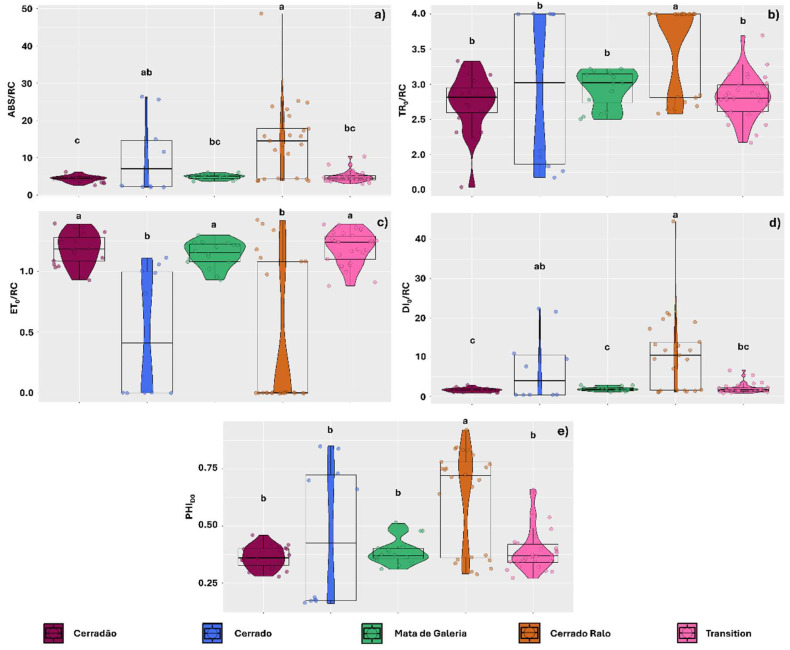
Chlorophyll *a* fluorescence parameters observed in thalli of the lichens *Parmotrema tinctorum* and *Usnea barbata* sampled across different landscapes in Emas National Park, Brazil. The parameters include: specific light absorption flux per reaction center (ABS/RC) (**a**); energy flux captured per reaction center at *t* = 0 (TR_0_/RC) (**b**); electron transport flux per reaction center (ET_0_/RC) (**c**); specific energy dissipation flux at the level of the antenna complex chlorophylls (DI_0_/RC) (**d**); and photosynthetic quenching associated with oxygen dissipation (PHI_DO_) (**e**). Violin plots depict data dispersion, with individual points representing observations. Boxplot central lines indicate medians, and letters above the boxplots denote means that do not differ significantly according to Tukey’s test (*p* < 0.05).

The landscape affected the amount of energy absorbed per reaction center (ABS/RC), with thalli sampled in Cerradão showing a more balanced energy absorption among reaction centers (4.34), with mean values similar to those observed in Mata de Galeria and transitional forest thalli (4.85 and 4.88, respectively) (Figure 1a). The TR_0_/RC mean values indicated that excited electrons were being transferred more slowly through the electron transport chain in thalli sampled in Cerrado Ralo (3.59), suggesting inefficiency or photosynthetic stress in the algal component (Figure 1b). However, electron transport, as indicated by ET_0_/RC mean values, was more efficient in thalli sampled in this environment and in Cerrado areas (0.38 and 0.48, respectively) (Figure 1c). DI_0_/RC data, however, were consistent with ABS/RC observations, showing that despite the efficient electron transport in thalli from Cerrado Ralo and Cerrado, high amounts of energy were dissipated as heat (7.39 and 10.93, respectively) (Figure 1d). Similarly, the high PHI_DO_ values observed in thalli from Cerrado Ralo and Cerrado (0.62 and 0.46, respectively) indicate the activation of excess energy dissipation mechanisms (Figure 1e).

**Figure 2:**
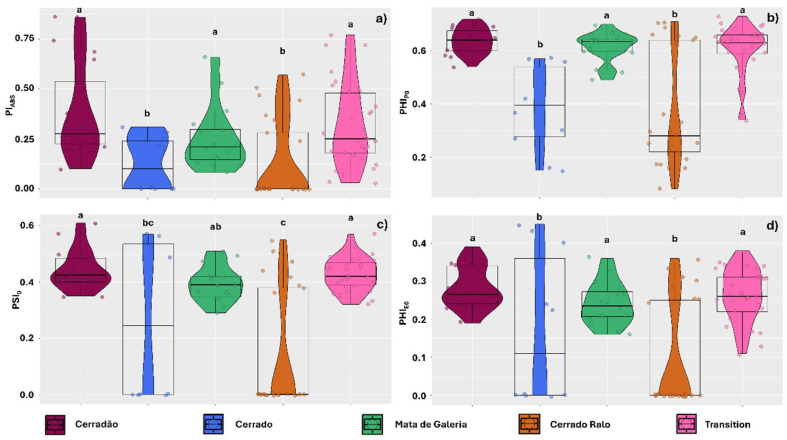
Chlorophyll *a* fluorescence parameters observed in thalli of the lichens *Parmotrema tinctorum* and *Usnea barbata* sampled across different landscapes in Emas National Park, Brazil. Parameters include photosynthetic performance index (Pi_ABS_) (**a**); maximum quantum yield of primary photochemistry (PHI_PO_) (**b**); probability of a trapped exciton transferring an electron beyond quinone in the electron transport chain (PSI_0_) (**c**); and quantum yield of electron transport (PHI_E0_) (**d**). Violin plots depict data dispersion, with individual points representing observations. Boxplot central lines indicate medians, and letters above the boxplots denote means that do not differ significantly according to Tukey’s test (*p* < 0.05).

Photosynthetic performance variables were also affected by the landscape, responding consistently with the previously analyzed energy transport variables. Photosynthetic performance (Pi_ABS_) was lower in thalli sampled in Cerrado and Cerrado Ralo (0.124 and 0.128, respectively) compared to those in the other landscapes of the park (0.24, 0.31, and 0.38 for Gallery Forest, Transition Forest, and Cerradão, respectively) (Figure 2a). Similarly, thalli sampled in Cerrado and Cerrado Ralo exhibited a lower maximum quantum yield of primary photochemistry (PHI_PO_) (0.38 and 0.37, respectively) than those from the other landscapes (Figure 2b). The probability of a trapped exciton moving an electron through the electron transport chain beyond quinone (PSI_0_) and the quantum yield of electron transport (PHI_E0_) were also similarly affected by the landscape, with lower values observed in Cerrado and Cerrado Ralo (0.26 and 0.14 for PSI_0_, and 0.17 and 0.09 for PHI_E0_, respectively) (Figure 2c,d).

**Figure 3:**
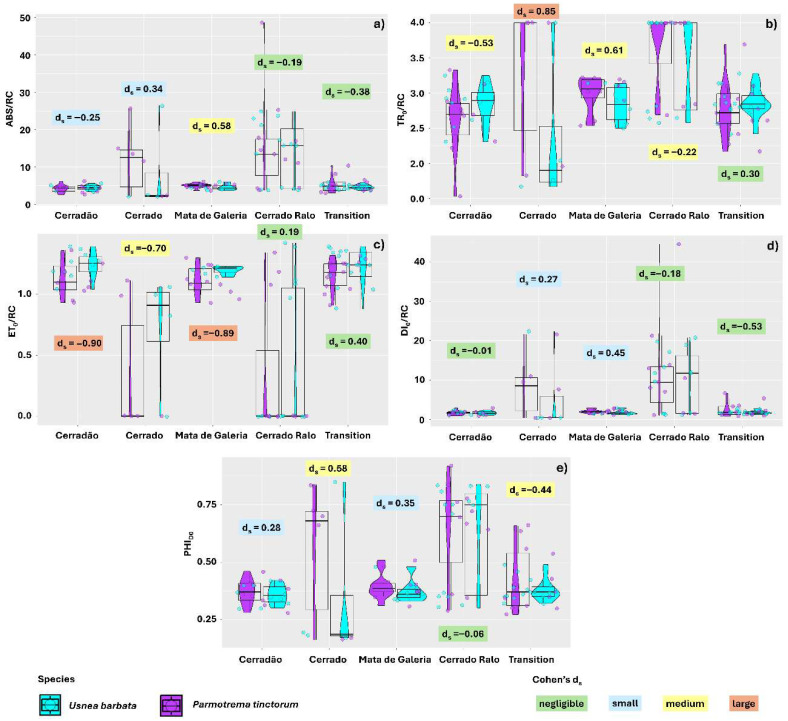
Effect size (Cohen’s d_s_ index) for chlorophyll *a* fluorescence parameters comparing thalli of the lichens *Parmotrema tinctorum* and *Usnea barbata* sampled across different landscapes in Emas National Park, Brazil. Parameters include: specific light absorption flux per reaction center (ABS/RC) (**a**); energy flux captured per reaction center at *t* = 0 (TR_0_/RC) (**b**); electron transport flux per reaction center (ET_0_/RC) (**c**); specific energy dissipation flux at the level of the chlorophylls in the antenna complex (DI_0_/RC) (**d**); and photosynthetic quenching involving oxygen dissipation (PHI_DO_) (**e**). The points associated with the violin plots represent data dispersion. The central line in the boxplots indicates the median, while the values and colors above the boxplots illustrate the Cohen’s d_s_ effect size.

When evaluating the effect of lichen species within the different landscapes analyzed, we found that for ABS/RC, the effect size was moderate only in Gallery Forest, where *U. barbata* (4.60) exhibited lower mean ABS/RC values than *P. tinctorum* (5.01). In the other areas, the effect size was negligible or small (Figure 3a). For TR_0_/RC, the effect was negligible only when comparing species sampled in Transition Forest. In Cerradão, Gallery Forest, and Cerrado Ralo, the effect size was moderate, with *U. barbata* showing lower mean values in Cerrado Ralo and Gallery Forest (2.84 and 3.53, respectively) compared to *P. tinctorum* (3.00 and 3.67). Similarly, *U. barbata* exhibited lower TR_0_/RC means in thalli sampled in Cerrado (2.37 and 3.30 for *U. barbata* and *P. tinctorum*, respectively), with a large effect size. However, in the Cerradão landscape, *P. tinctorum* showed lower TR_0_/RC mean values (2.63) than *U. barbata* (2.85) (Figure 3b).

The ET_0_/RC values were also influenced by species, with *P. tinctorum* showing higher means than *U. barbata* across all landscapes evaluated. However, while the effect size was negligible in the Transition Forest and Cerrado Ralo, it was considered large in the Cerradão and Gallery Forest (Figure 3c). For DI_0_/RC, the differences between species were negligible in Cerradão, Cerrado Ralo, and Transition Forest. In Cerrado and Gallery Forest, the effect size was small, with *U. barbata* showing lower mean energy dissipation as heat (5.88 and 1.76, respectively) compared to *P. tinctorum* (8.40 and 1.96, respectively) (Figure 3d). Differences in PHI_DO_ means were negligible between species in Cerrado Ralo and small in Cerradão and Gallery Forest. These results corroborate previous findings, with *U. barbata* exhibiting lower energy dissipation means (0.35 and 0.38, respectively) than *P. tinctorum* (0.37 and 0.39, respectively) (Figure 3e).

**Figure 4:**
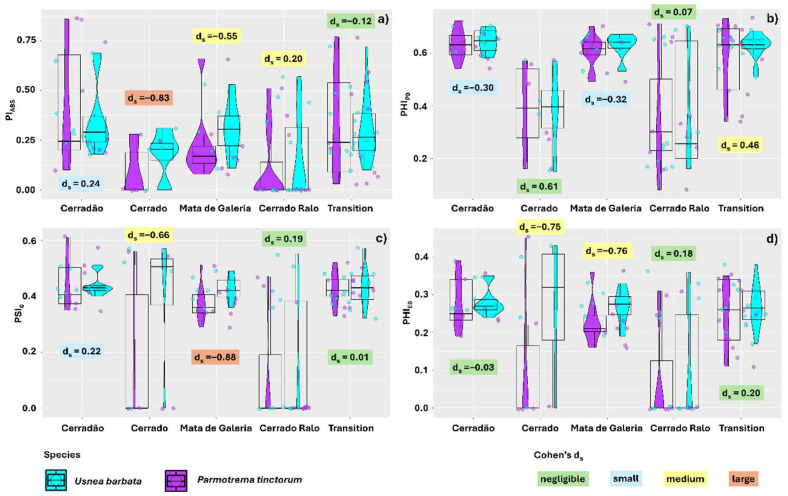
Effect size (Cohen’s d_s_ index) for chlorophyll *a* fluorescence parameters comparing thalli of the lichens *Parmotrema tinctorum* and *Usnea barbata* sampled across different landscapes in Emas National Park, Brazil. Parameters include photosynthetic performance index (Pi_ABS_) (**a**); primary photochemical maximum quantum yield (PHI_PO_) (**b**); probability of a trapped exciton moving an electron through the electron transport chain after Quinone (PSI_0_) (**c**); and electron transport quantum yield (PHI_E0_) (**d**). The points associated with the violin plots represent data dispersion. The central line in the boxplots indicates the median, while the values and colors above the boxplots illustrate the Cohen’s d_s_ effect size.

Photosynthetic performance (Pi_ABS_) of the thalli was also species-dependent, with the effect size being negligible only for thalli sampled in Transition Forest. In Cerradão, the effect size was small, with *U. barbata* showing a higher average performance (0.44) than *P. tinctorum* (0.37). In the Gallery Forest and Cerrado Ralo landscapes, the results were similar, with *U. barbata* exhibiting better photochemical performance (0.30 and 0.11, respectively) compared to *P. tinctorum* (0.23 and 0.15, respectively); however, the effect size was medium. In the Cerrado area, the effect size was large, and the results were consistent in the comparison between the species (0.18 and 0.09, respectively, for *U. barbata* and *P. tinctorum*) (Figure 4a).

The PHI_PO_ data were also differentially affected by lichen species, although the differences were negligible in the Cerrado and Cerrado Ralo areas. In the Cerradão and Gallery Forest areas, however, the effect size was small, with *U. barbata* thalli showing better quantum yield (0.64 and 0.62, respectively) than *P. tinctorum* (0.63 and 0.61, respectively). In the Transition Forest, however, the effect size was moderate, with *U. barbata* (0.62) showing a higher average yield than *P. tinctorum* (0.58) (Figure 4b). For the PSI_0_ data, the effect size was negligible in thalli sampled from the Cerrado Ralo and Transition Forest. In the Cerradão landscape, however, the effect was small, with the highest averages found in *U. barbata* (0.46 and 0.43 for *U. barbata* and *P. tinctorum*, respectively). In the Cerrado area, the effect size was moderate, but the results were similar (0.40 and 0.18 for *U. barbata* and *P. tinctorum*, respectively). Higher averages for *U. barbata* (0.43) compared to *P. tinctorum* (0.38) were also found in the Gallery Forest landscape, though the effect size was large (Figure 4c).

The electron transport quantum yield (PHI_E0_) was also affected by species in the different landscapes evaluated. However, the effect size was negligible for samples from Cerradão, Cerrado Ralo, and Transition Forest. In the Cerrado and Gallery Forest landscapes, the effect size was moderate, with *U. barbata* showing higher average yields (0.40 and 0.27, respectively) compared to *P. tinctorum* (0.23 and 0.18) (Figure 4d).

**Figure 5:**
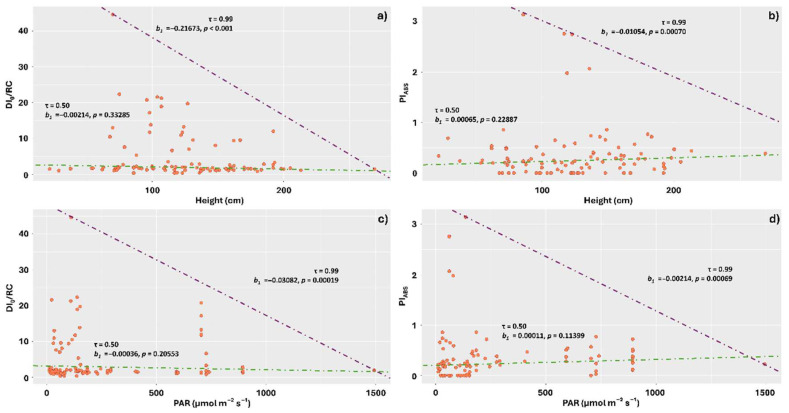
Quantile regression analysis of the relationships between chlorophyll *a* fluorescence parameters DI_0_/RC and Pi_ABS_ with the sampling height of *Parmotrema tinctorum* and *Usnea barbata* lichens on tree trunks and photosynthetically active radiation (PAR) observed in different landscapes of Emas National Park, Brazil. Specific energy dissipation flux at the antenna complex chlorophylls level (DI_0_/RC) and photosynthetic performance index (Pi_ABS_) as a function of sampling height (**a**,**b**); DI_0_/RC and Pi_ABS_ as a function of PAR (**c**,**d**). The figures show triangle-shaped envelopes corresponding to 0.50 (green line) and 0.99 (purple line) quantile fits.

When we evaluated the effect of lichen sampling height on tree trunks, PAR, and different components of the visible spectrum on DI_0_/RC and Pi_ABS_, the regression at the 0.99 quantile indicated a negative effect of increasing lichen sampling height on energy dissipation in the form of heat, with thalli sampled on trunks between heights of 70 and 130 cm tending to show the highest average dissipation (Figure 5a). Similarly, this quantile showed a negative effect of increasing height on the photosynthetic performance index (Pi_ABS_), with the highest performance concentrated in the height range of 60 to 180 cm (Figure 5b).

At quantile 0.99, PAR also affected DI_0_/RC and Pi_ABS_, with the greatest dissipation and performances observed under lower amounts of PAR (respectively, between 0 and 200 µmol m^−2^ s^−1^ for DI_0_/RC, and between 0 and 300 µmol m^−2^ s^−1^ for Pi_ABS_) (Figure 5c,d).

**Figure 6:**
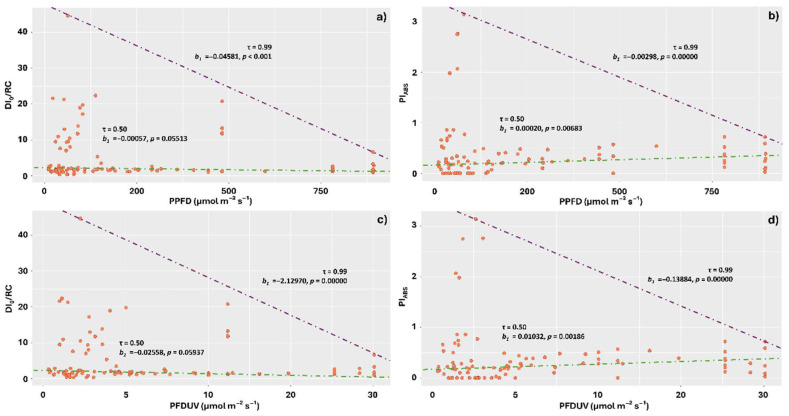
Quantile regression analysis of the relationships between chlorophyll *a* fluorescence parameters DI_0_/RC and Pi_ABS_ with the components of the visible spectrum: photosynthetic photon flux density (PPFD) and ultraviolet photon flux density (PFDUV), incident on thalli of *Parmotrema tinctorum* and *Usnea barbata* lichens sampled in different landscapes of Emas National Park, Brazil. Specific energy dissipation flux at the level of the antenna complex chlorophylls (DI_0_/RC) and photosynthetic performance index (Pi_ABS_) as a function of PPFD (**a**,**b**); DI_0_/RC and Pi_ABS_ as a function of PFDUV (**c**,**d**). The figures show triangle-shaped envelopes corresponding to 0.50 (green line) and 0.99 (purple line) quantile fits.

At quantile 0.99, increasing PPFD on the stems negatively affected energy dissipation in the form of heat, with the highest average dissipations concentrated in the range between 30 and 500 µmol m^−2^ s^−1^ (Figure 6a). The effects of PPFD on Pi_ABS_, however, were contrasting for quantiles 0.50 and 0.99. At 0.50, the relationship was positive, with increases in PPFD enhancing photochemical performance, consistent with the observations for DI_0_/RC. At 0.99, however, the relationship was negative, with increases in PPFD leading to decreased performance in the stems. The highest performances were observed in the range between 0 and 120 µmol m^−2^ s^−1^ (Figure 6b).

Similar results were observed for the PFDUV component. At quantile 0.99, increases in PFDUV led to reductions in DI_0_/RC, while at quantile 0.50, increases positively affected photosynthetic performance. At quantile 0.99, however, higher PFDUV values negatively affected performance, with the highest indices observed in the range between 1 and 4 µmol m^−2^ s^−1^ for this component (Figure 6c,d).

**Figure 7:**
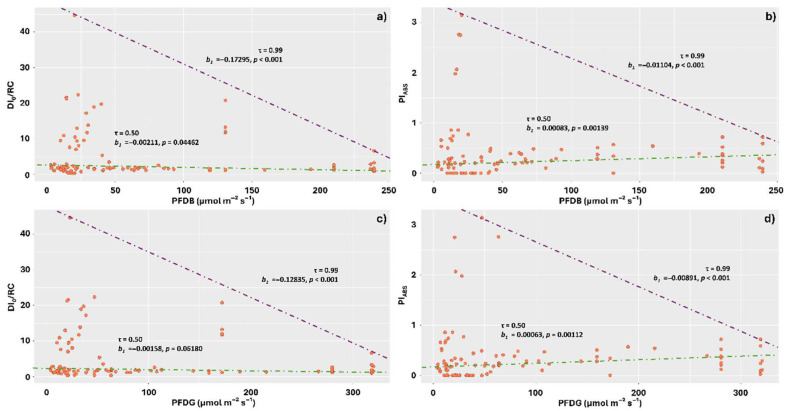
Quantile regression analysis of the relationships between chlorophyll *a* fluorescence parameters DI_0_/RC and Pi_ABS_ with the visible spectrum components: blue photon flux density (PFDB) and green photon flux density (PFDG), incident on thalli of *Parmotrema tinctorum* and *Usnea barbata* lichens sampled in different landscapes of Emas National Park, Brazil. Specific energy dissipation flux at the level of the antenna complex chlorophylls (DI_0_/RC) and photosynthetic performance index (Pi_ABS_) as a function of PFDB (**a**,**b**); DI_0_/RC and Pi_ABS_ as a function of PFDG (**c**,**d**). The figures show triangle-shaped envelopes corresponding to 0.50 (green line) and 0.99 (purple line) quantile fits.

The means of PFDB and PFDG affected the DI_0_/RC values in a similar way, with negative relationships observed in the quantile triangle from 0.50 to 0.99. Thus, increases in these visible light components negatively affected energy dissipation, indicating photochemical stress in the stems where the incidence of these light components was lower. The highest energy dissipation in the form of heat was observed in the ranges of 0 to 50 µmol m^−2^ s^−1^ for PFDB and 0 to 60 µmol m^−2^ s^−1^ for PFDG (Figure 7a,c).

The Pi_ABS_ responses were also similar for PFDB and PFDG, with contrasting responses observed between the 0.50 and 0.99 quantiles. In the first quantile, Pi_ABS_ means tended to increase in response to increases in PFDB and PFDG values, which corroborates the results observed for DI_0_/RC. At the 0.99 quantile, however, photosynthetic performance decreased in response to increased values of the aforementioned components (Figure 7b,d).

**Figure 8:**
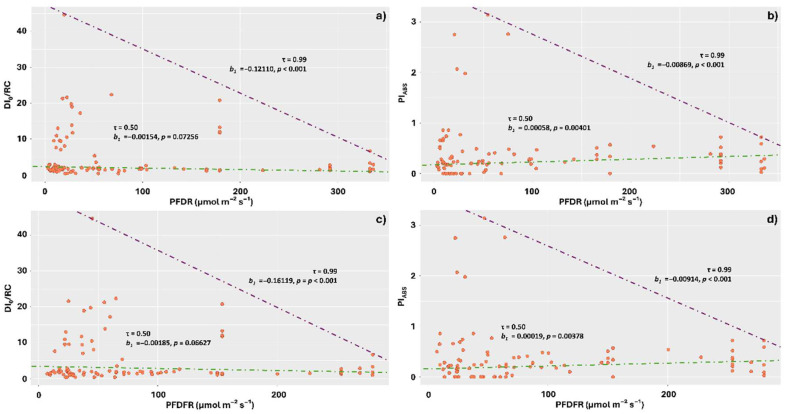
Quantile regression for the relationships between chlorophyll *a* fluorescence parameters (DI_0_/RC and Pi_ABS_) and the visible spectrum components: photon flux density red (PFDR) and photon flux density far-red (PFDFR), incident on thalli of the lichens *Parmotrema tinctorum* and *Usnea barbata* sampled in different landscapes of the Emas National Park, Brazil. Specific energy dissipation flux at the level of the antenna complex chlorophylls (DI_0_/RC) and photosynthetic performance index (Pi_ABS_) as a function of PFDR (**a**,**b**); DI_0_/RC and Pi_ABS_ as a function of PFDFR (**c**,**d**). The figures show triangle-shaped envelopes corresponding to 0.50 (green line) and 0.99 (purple line) quantile fits.

The DI_0_/RC averages also responded negatively to the increase in the amounts of the red component of visible light, indicating higher stress levels under lower incidences of PFDR, with the highest averages concentrated in the range of 0 to 70 µmol m^−2^ s^−1^ (Figure 8a). Pi_ABS_, however, exhibited a response that corroborates this result at the 0.50 quantile level, with the lowest performances observed at the lowest incidences of PFDR. At the 0.99 quantile, however, the response was opposite, with Pi_ABS_ values decreasing as the amount of this component increased (Figure 8b).

The responses to far-red light were similar to those observed for PFDR, with DI_0_/RC averages tending to decrease in response to increasing PFDFR at the 0.99 quantile (Figure 8c), with the greatest dissipations concentrated in the ranges of 10 to 70 µmol m^−2^ s^−1^. The results observed for Pi_ABS_ at the 0.50 quantile corroborate those observed for DI_0_/RC; however, at the 0.99 quantile, they show a reduction in photochemical performance as the incident levels of PFDFR increased (Figure 8d).

**Figure 9:**
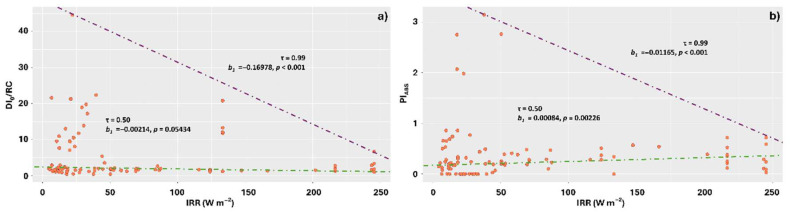
Quantile regression for the relationships between chlorophyll *a* fluorescence parameters (DI_0_/RC and Pi_ABS_) and irradiance (IRR), incident on thalli of the lichens *Parmotrema tinctorum* and *Usnea barbata* sampled in different landscapes of the Emas National Park, Brazil. Specific energy dissipation flux at the level of the antenna complex chlorophylls (DI_0_/RC) and photosynthetic performance index (Pi_ABS_) as a function of IRR (**a**,**b**).

Irradiance significantly affected the DI_0_/RC averages only at the 0.99 quantile, with lower IRR values leading to greater dissipation, with these being concentrated in the range of 0 to 50 W m^−2^ (Figure 9a). Pi_ABS_ values, on the other hand, exhibited contrasting responses for the different quantiles. At 0.50, the best performances were observed under higher irradiances; however, at 0.99, the highest Pi_ABS_ averages were observed under lower IRRs (Figure 9b).

## 3. Discussion

### 3.1. High Levels of Energy Dissipation Observed in Thalli Sampled from the Cerrado and Cerrado Ralo Landscapes Resulted in Lower Photochemical Performance

In the Cerrado, thalli were exposed to excessive light conditions (54,130 lm m^−2^), which can cause photoinhibition, burns, and dehydration [32]. These high light intensities led to the activation of algal protective mechanisms, including energy dissipation in the form of heat. Osyczka and Myśliwa-Kurdziel [1] suggest that the functional plasticity of the thylakoid membranes in photobionts plays a significant role in the dispersal abilities of lichens in particular habitats. *U. barbata* and *P. tinctorum* are abundant in Cerrado areas, and our findings support the idea that these species possess efficient adaptive and photoprotective mechanisms, which ensure their resilience and help prevent photodamage. 

The photobionts of *U. barbata* and *P. tinctorum* are green algae of the genus *Trebouxia* [33,34,35,36], which are nonmotile coccoid algae with characteristic pyrenoids and lobed chloroplasts [37]. Studies show that the photochemical efficiency of these algae can respond differentially to environmental changes, such as increasing temperatures. For example, *Trebouxia asymmetrica* did not alter its F_v_/F_m_ values when acclimated to different temperatures (17 and 20 °C). In contrast, *Trebouxia* sp. ‘*arnoldoi*’ and *T. cretacea* were more sensitive to temperature changes, with a decrease in F_v_/F_m_ when exposed to 17 °C [38]. Thus, the photochemical and photosynthetic performance of thalli can be influenced by the symbiotic *Trebouxia* species, as these algae exhibit varying sensitivities to fluctuations in temperature and irradiance in their growth environment [39].

In this study, we examined the sensitivity of the photobionts of *U. barbata* and *P. tinctorum* to high light levels in the open Cerrado physiognomy. High irradiation levels can induce oxidative stress in photobionts. Muhetaer et al. [40] demonstrated a significant correlation between increased PAR and elevated activities of antioxidant enzymes, as well as higher H_2_O_2_ content, in the algae *Phormidium ambiguum* and *Microcystis aeruginosa*. Non-photochemical quenching (NPQ) in the chloroplast helps to dissipate excess light energy from PSII, constituting one of the mechanisms that protect photobionts against oxidative stress [41,42]. However, despite the protective role of NPQ, inevitable damage to PSII leads to a reduction in linear electron transport, which in turn limits downstream carbon assimilation. 

In the Cerrado Ralo landscape, where irradiation levels are high (47,666 lm m^−2^), we also observed the activation of photoprotective mechanisms in the thalli. These mechanisms acted by increasing energy dissipation and decreasing chlorophyll *a* fluorescence performance. This is because the canopy in this plant formation is discontinuous, with many open areas that allow light to pass through and directly impact the thalli. Increases in NPQ or energy dissipation via active reaction centers (DI_0_/RC) are efficient mechanisms to alleviate excitation pressure in PSII, reducing damage to the photosynthetic apparatus [43]. However, this comes at the cost of overall photosynthetic performance (Pi_ABS_) [27]. Several studies highlight the negative effects of high light exposure on Pi_ABS_ [44,45].

As expected for species exposed to excessive light, the high light intensity in the thalli of *U. barbata* and *P. tinctorum* in the Cerrado and Cerrado Ralo areas led to an increase in the average ABS/RC. This was due to a reduction in antenna size (fewer light-harvesting pigments—chlorophylls and carotenoids—per reaction center), which subsequently decreased ET_0_/RC. With reduced QA, which cannot be oxidized efficiently, the energy is not used effectively in photochemical reactions and electron transport but instead dissipates as heat (resulting in increased DI_0_/RC) [27,44,46].

### 3.2. U. barbata Thalli Exhibited Superior Photochemical Performance Compared to P. tinctorum Across All Sampled Landscapes

Studies suggest that *Trebouxia* photobionts possess mechanisms to repair PSII damage and resynthesize damaged proteins, primarily through the stimulation of cyclic electron transport (CET) in PSI. Beckett et al. [47] suggested that CET contributes to the photoprotection of lichenized algae under light stress. CET promotes ATP synthesis and facilitates the rapid recovery of PSII [43,48]. Although both *U. barbata* and *P. tinctorum* may activate these pathways, *U. barbata* exhibits additional protective mechanisms that enhance its resilience across different landscapes. One key factor contributing to *U. barbata*’s higher resistance is its production of antioxidant metabolites, particularly usnic acid, which protects against oxidative stress caused by excessive light [49]. As the dominant secondary metabolite in *Usnea*, usnic acid has strong antioxidant and cytoprotective properties [50]. Moreover, *Usnea* species retain little water [51], yet their photosynthetic pathways reactivate almost immediately upon exposure to humid air, demonstrating high plasticity in response to water deficits [52].

The morphological structure of usnic hair lichens further supports photoprotection. Their light-colored, semi-transparent cortex allows light penetration even when dry, exposing desiccated chlorophylls to oxidative stress. The yellow-green pigmentation, a combination of chlorophyll and usnic acid, helps filter solar radiation by reflectance, reducing heat stress [53,54,55] and prolonging photosynthesis after hydration [52]. Usnic acid accumulates in the cortical layer of *Usnea* lichens, acting as a natural light filter that regulates solar irradiance reaching the algal layer and protects the photobiont from excessive radiation [56]. This ability to synthesize usnic acid contributes to *Usnea*’s physiological plasticity. For instance, *U. barbata* contains approximately 1.5% usnic acid [57], which enhances light reflection and functions as a flexible solar radiation screen [58]. Phenolic acids also contribute to energy dissipation and protection against free radicals [49], acting in concert with the branched architecture of the *Usnea* thallus to minimize direct exposure of the photobiont to intense radiation. Engel [59] suggests that in addition to usnic acid, the metabolites atranorin, chloroatra-norin, barbatolic acid, lobaric acid, and salazinic acid, present in the thalli of lichens of this genus, have protective action against UVB radiation.

Gauslaa and Goward [23] suggest that under drought conditions, the cortical parenchyma of usnic lichens shrinks, leading to the collapse of photobiont cells. As a result, air fills the cortex, making it partially transparent. These morphological changes cause the cortical window to close partially, increasing chlorophyll reflectance and enhancing thallus photoprotection. The photoprotection responses observed in *U. barbata* across different environments can thus be attributed to the combined effects of high light incidence and low water availability. In contrast, under high humidity and shaded conditions, the cortex expands, and the photobiont cells regain turgidity, rendering the cortex more transparent. This shift optimizes photosynthesis by enhancing light capture.

Despite differences between *U. barbata* and *P. tinctorum*, species of *Parmotrema* are also considered resilient. Its widespread distribution across tropical phytophysiognomies highlights its ability to withstand low humidity and high light conditions. Studies show that in *Usnea* thalli, but especially in foliose lichens such as those of the genus *Parmotrema* [60], atranorin, a secondary metabolite belonging to the depsid class, contributes to the photoprotection of the thallus. Atranorin can prevent water from entering the spaces between hyphae in the cortex. The air-filled cavities with white atranorin crystals reflect excess light, playing a significant photoprotective role for the symbiotic green algae [54]. Despite this, the fruticose growth form of *U. barbata*, characterized by the presence of a cartilaginous central axis and a denser medulla [19,61], together with the production of high concentrations of cortical usnic acid, likely provides a more effective photoprotective effect than the atranorin deposited in the foliose cortex of *P. tinctorum*.

The more pronounced photoinhibition of photosynthesis in *P. tinctorum* appears to be a long-term regulatory mechanism for PSII, reducing metabolism and enhancing resistance to prolonged desiccation [4]. Barták et al. [62] suggest that lichens such as *Xanthoria elegans* tolerate high light stress effectively due to photoprotective mechanisms activated during photoinhibition. These mechanisms include photoinhibitory quenching (qIₜ), which involves structural modifications in the photosynthetic apparatus and constitutes a major component of NPQ. Similarly, Rautenberger and Hurd [63] observed that the combined action of photoinhibitory quenching and PSII reaction center quenching in response to light stress is a key mechanism enabling the chlorophyte *Ulva rigida* to thrive and form green tides in coastal ecosystems.

Although *P. tinctorum* exhibited lower Pi_ABS_ levels, its capacity for heat dissipation was comparable to that of *U. barbata*, suggesting an efficient NPQ mechanism. Miyake et al. [64] suggested that, in desiccated thalli of *P. tinctorum*, the dissipation of excess light energy is characterized by a rapid fluorescence decay with a time constant of 27 ps in the far-red region. This quenching mechanism exhibits extremely high efficiency and is likely associated with the formation of a rapid quenching state in the peripheral-antenna system of PSII during desiccation. On the other hand, Adams et al. [65] demonstrated that green algal photobionts from lichens inhabiting arid environments accumulate zeaxanthin in response to increasing sunlight intensity, confirming the xanthophyll cycle-dependent nature of NPQ in symbiotic green algae. Their findings also indicated that substantial light exposure, beyond desiccation alone, is required to induce zeaxanthin accumulation in these photobionts. Therefore, the specific contribution of the xanthophyll cycle to fluorescence quenching in *P. tinctorum* remains an open question.

### 3.3. Lichens Sampled Higher on the Stem and Exposed to Higher PAR Levels Exhibited Lower Energy Dissipation as Heat and Reduced Photochemical Performance

High radiation levels induce photochemical damage, necessitating mechanisms to eliminate reactive oxygen species (ROS). If these mechanisms are compromised by other environmental stressors, excessive ROS accumulation can damage the photosynthetic apparatus, particularly PSII [66,67]. Although NPQ is only one of a host of mechanisms that protect against high light, our findings indicate that lichens sampled at higher positions on the stem—where they were more exposed to radiation in the open Cerrado and Cerrado Ralo landscapes—exhibited insufficient dissipation mechanisms, leading to photoinhibition and reduced photosynthetic capacity in photobiont cells. Mkhize et al. [68] suggest that lichens growing in fluctuating light conditions generally exhibit higher levels of NPQ compared to those exposed to constant full sunlight. If the lichens located further down the stem are subjected to more variable light conditions than those higher up, this could explain their elevated NPQ values. However, in the evaluated species, this damage appears to be reversible as long as the stress is not prolonged [11]. Additionally, contrasting performance responses at the 0.50 and 0.99 quantile levels suggest that photoprotective mechanisms are only activated beyond certain radiation thresholds, ultimately reducing Pi_ABS_.

### 3.4. Increasing Intensities of the Different Spectrum Components Reduced DI_0_/RC and Increased Pi_ABS_

Interestingly, when evaluating the spectral components separately, we observed a positive photochemical response of the thalli to individual increases in each component’s intensity. This is because, in general, a reduction in DI_0_/RC indicates that less energy is being dissipated per reaction center, meaning that more energy is effectively used for electron transport, which is favorable for photosynthetic efficiency. This suggests good acclimation of the lichen species to the prevailing environmental conditions (e.g., [69]). Conversely, an increase in Pi_ABS_ indicates that the photosynthetic apparatus is more efficient and functionally active, with a greater capacity to convert absorbed light into chemical energy. This reflects a well-performing photosynthetic system adapted to environmental conditions [70]. This finding highlights the photochemical resistance of *P. tinctorum*, and especially *U. barbata*, to increased light levels, a resilience likely ensured by their ability to effectively quench excess energy under stress.

Numerous studies have demonstrated highly efficient NPQ mechanisms in lichens under high light intensity [62]. Barták et al. [71] show an increase in NPQ with desiccation of lichen thalli. In lichens that have dried out, NPQ is characterized by rapidly decaying fluorescence components around 690 and 740 nm. Notably, fluorescence quenched under dry conditions recovers within minutes after rewetting, suggesting that most quenching results from a reversible change in the state of antenna proteins rather than a reduction in protein content [72]. Beckett et al. [11] demonstrated that in desiccated thalli, the formation of ROS can lead to photoinhibition and photo-oxidative stress, thereby reducing the carbon fixation capacity of photobionts. Tolerance can be achieved by minimizing ROS production, through the synthesis of antioxidant pigments, thermal dissipation of excess absorbed light energy, scavenging of ROS after formation, or repairing ROS-induced damage. Evidence indicates the presence of two distinct pathways for dissipating excess light energy in dry thalli of *P. tinctorum*: one characterized by a rapid fluorescence decay with a time constant of 27 ps in the far-red region, absent in wet thalli, and another involving accelerated fluorescence decay in the 685–700 nm spectral range [64].

Field measurements during the dry season revealed that among the 11 macrolichen species evaluated, only the foliose chlorolichen *P. tinctorum* remained metabolically active, exhibiting slight carbon assimilation even under desiccation. This finding highlights its remarkable resistance to extreme environments [73]. Studies indicate that in *Trebouxia* algae exposed to desiccation and high light, variable chlorophyll fluorescence is lost, signifying a suspension of charge separation in PSII. Simultaneously, basal fluorescence (F0) is strongly quenched, which has been interpreted as evidence of high photoprotective non-radiative dissipation (NRD) [74]. This mechanism likely explains the observed resistance of *P. tinctorum* and *U. barbata* to increasing spectral components. Understanding these adaptive physiological strategies provides essential insights into the survival and distribution of lichens in the stressful landscapes of the savanna, forming a strong foundation for future research in this field.

## 4. Materials and Methods

**Figure 10:**
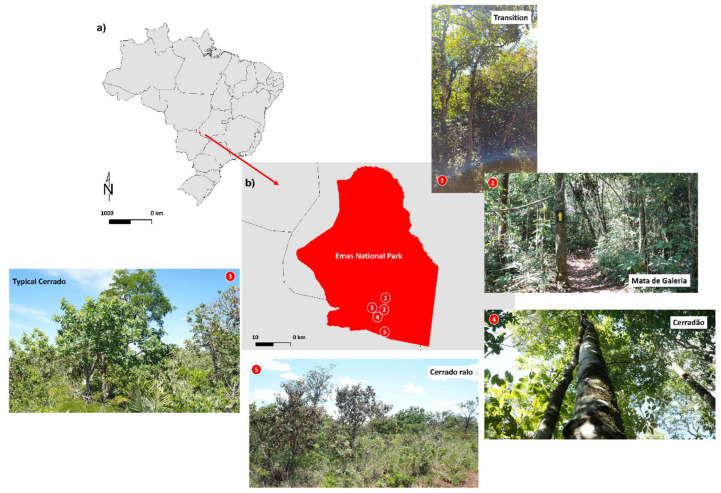
(**a**) Location of Emas National Park in the state of Goiás, Brazil. (**b**) Distribution of the different sampled landscapes within the park. The photographs depict the characteristic vegetation formations of each landscape type.

The foliose lichen *Parmotrema tinctorum* and the fruticose lichen *Usnea barbata* were evaluated in this study. Samples were observed in situ at their natural occurrence sites, which included different landscape types within the Emas National Park, Goiás, Brazil (Figure 10a). These landscapes were classified as Cerradão, Cerrado, Cerrado Ralo, Mata de Galeria, and Mata de Transição (Transition) based on the characteristics of the resident vegetation, following Lopes-Assad [75], and considering local light availability.

- Cerradão (18°15′05.2″ S; 52°53′12.2″ W)—Mean illuminance: 13,023 lm m^−2^. Characterized by dense forest vegetation with structural features intermediate between savanna and tropical forest. Dominated by a continuous tree stratum, emergent trees, and a relatively open understory.
- Typical Cerrado (18°19′42″ S; 52°52′51″ W)—Mean illuminance: 54,130 lm m^−2^. An open savanna formation with predominantly herbaceous and grassy vegetation, interspersed with a few shrubs and sparse trees.
- Mata de Galeria (Gallery Forest) (18°15′33.6″ S; 52°53′13.7″ W)—Mean illuminance: 26,967 lm m^−2^. Located along the banks of the Formoso River, this vegetation type is dense, with multiple layers of trees, shrubs, and epiphytes.
- Cerrado Ralo (18°15′33.4″ S; 52°53′21.6″ W)—Mean illuminance: 47,666 lm m^−2^. Slightly more open than the typical Cerrado, with a predominance of herbaceous vegetation, sparse shrubs, and widely spaced trees.
- Mata de Transição (Transition) (18°13′44.1″ S; 52°52′40.8″ W)—Mean illuminance: 17,010 lm m^−2^. Represents an intermediate stage between open areas (campo sujo) and forested formations (cerradão). The vegetation is mixed, consisting of trees, shrubs, and grasses. The canopy is discontinuous, with sparse trees and a well-developed herbaceous-shrub layer (Figure 10b).

At each location, lichen sampling was conducted within a physically demarcated 200 m^2^ area.

The average illuminance (lm m^−2^) levels in each landscape were determined from measurements taken at five randomly selected points within the sampling area. Data were collected using a Li-Cor LI-180 spectroradiometer on 5–7 November. Physiological parameters and incident light quality on the lichen thalli were recorded between 10:00 and 11:00 AM. Measurements were taken in situ, with lichens remaining attached to tree trunks while the spectroradiometer was positioned accordingly. Based on these measurements, light intensity across landscapes was classified as low (<900 lm m^−2^—equivalent to <16.65 µmol m^−2^ s^−1^), medium (900–23,000 lm m^−2^—equivalent to 16.65–425.50 µmol m^−2^ s^−1^), high (23,000–50,000 lm m^−2^—equivalent to 425.50–925.00 µmol m^−2^ s^−1^), or very high (>50,000 lm m^−2^—equivalent to >925.00 µmol m^−2^ s^−1^), following an adaptation of the classification proposed by Nobel et al. [76].

### 4.1. Chlorophyll a Fluorescence in Thalli

Transient OJIP fluorescence of chlorophyll *a* was measured using a portable fluorometer (FluorPen FP 100, Photon Systems Instruments, Drásov, Czech Republic). Prior to measurement, all lichen thalli were dark-adapted for 30 min to ensure complete oxidation of the photosynthetic electron transport system. Subsequently, they were exposed to a 3000 µmol m^−2^ s^−1^ blue light pulse, and fluorescence kinetics were recorded. The minimum fluorescence (F_0_) was measured at 50 μs when all PSII reaction centers were open, defining the O step. This was followed by the J step (at 2 ms), the I step (at 30 ms), and the maximum fluorescence (F_m_), corresponding to the P step when all PSII reaction centers were closed. These fluorescence parameters were used to estimate various PSII bioenergetic indices, following Strasser et al. [77], including: ABS/RC—specific light absorption flux per reaction center; TR_0_/RC—energy flux captured per reaction center at *t* = 0; ET_0_/RC—electron transport flux per reaction center; DI_0_/RC—specific energy dissipation flux at the antenna chlorophyll level; PHI_PO_—primary photochemical maximum quantum yield; PSI_0_—probability, at *t* = 0, of a trapped exciton moving an electron through the electron transport chain after Quinone; PHI_E0_—electron transport quantum yield after dark adaptation (30 min); PHI_DO_—photosynthetic quenching involving oxygen dissipation; and Pi_ABS_—photosynthetic performance index, integrating energy transfer processes from initial absorption to PQ reduction.

### 4.2. Light Measurement

The different types of light incident on the stems were measured using a spectroradiometer model LI-180, Li-Cor. For this purpose, the equipment was positioned attached to the tree trunk, in the position where the lichen occurred. The equipment was moved towards the sun, in order to capture the direct incident light. The spectral composition of the light was recorded in wavelength intervals of 1 nm (bandwidth of 12 nm). The information was recorded for the interval 380 to 780 nm and the components of the visible spectrum classified in the bands: Photosynthetic Photon Flux Density (PPFD), which refers to the number of photons that promote photosynthesis (in the range of 400 to 700 nm) and that reach a surface; Photon Flux Density Ultraviolet (PFDUV), which refers to the photon flux that is in the ultraviolet (UV) band, with wavelengths below 400 nm. Photon Flux Density Blue (PFDB), which measures the amount of photons in the blue light range (usually between 400 and 500 nm). Photon Flux Density Green (PFDG), which refers to the photon flux in the green light range (between 500 and 600 nm). Photon Flux Density Red (PFDR), which measures the amount of photons in the red range (usually between 600 and 700 nm). Photon Flux Density Far-Red (PFDFR), which refers to the photon flux in the far-red light range (usually between 700 and 800 nm) and Irradiance (IRR), which refers to the total power of electromagnetic radiation (including visible and non-visible light) that reaches a surface. It is a measure of the total energy delivered by radiation, regardless of its usefulness for photosynthesis.

Information on photosynthetically active radiation (PAR) incident on the stems was obtained using a PAR Sensor (APG-SQ-316, Apogee, North Logan, UT, USA). This sensor consists of a bar with 6 sensors, self-powered with an output of 0 to 800 mV. The sensor was previously calibrated for use with sunlight and subsequently positioned attached to the tree trunk, at the height of the lichen to be sampled. PAR information was collected by positioning the sensor towards the sun, in order to capture the direct incident light.

### 4.3. Position of Lichen on the Trunk

The position of each lichen on the tree trunk was recorded based on its height (cm) above the ground. Height measurements were obtained using a millimeter tape, extended from the ground to the lichen’s location, with or without the aid of a ladder. The evaluated height range spanned from 0.22 to 269 cm.

### 4.4. Experimental Design and Statistical Analyses

In each landscape, an average of 20 lichen specimens per species were evaluated. Samples were randomly collected along a 30 m transect extending toward the center of the landscape. The transect started 3 m from the edge of each study area to minimize edge effects. We employed Generalized Linear Mixed Models (GLMMs) to assess the effects of Landscape, Species, lichen height on the stem, and light spectrum characteristics on chlorophyll *a* fluorescence. Prior to model construction, we tested for multicollinearity among the variables using the “Car” package [78] and excluded those with a variance inflation factor (VIF) >5. The explanatory variables included Landscape, Species, Height, Photosynthetic Photon Flux Density (PPFD), Photon Flux Density in the UV range (PFDUV), Green (PFDG), Red (PFDR), Far-red (PFDFR), Infrared Radiation (IRR), and Photosynthetically Active Radiation (PAR). Chlorophyll *a* fluorescence parameters were analyzed separately, with Species and Height included as random factors, while the remaining explanatory variables were treated as fixed factors.

We utilized the MCMCglmm package [79] to perform the analyses in a Bayesian framework with the Markov Chain Monte Carlo algorithm. A total of 80,000 iterations with 20,000 burn-in chains and a Gaussian distribution were used. The Akaike information criteria (AIC) were used to select the best model: the model with the smallest AICc (the AIC corrected for sample size and the number of parameters), which was considered to be the most plausible for the explanation of the observed patterns [80]. The Delta AICci (ΔAICci, where i represents each model) was calculated as the difference between the AICc for the ith model and the smallest AICc observed. We also determined Akaike’s weight (wAICc), which represents the relative contribution of the ith model to the explanation of the observed pattern, given a set of competing models. The models with ΔAICc < 2 were all considered equally plausible as explanations of the observed pattern [81]. At the end of the analysis, the significance of the different models was compared to the simplest evaluated model (Landscape + Species).

The data were subjected to a normality test and subsequently analyzed using one-way ANOVA to assess the effect of Landscape on chlorophyll *a* fluorescence parameters. Significant differences between sampling sites were determined using Tukey’s test (*p* < 0.05). Additionally, the effect size of Species within each sampled Landscape was quantified using Cohen’s *d*, calculated with the *cohensD* function from the “effsize” package [82]. The *d* values were classified as small (≤0.2), medium (>0.2 to ≤0.5), or large (>0.5), following Sullivan and Feinn [83]. The relationships between visible light spectrum components, PAR, and lichen height on the trunk with fluorescence parameters were tested using quantile regression [84], implemented via the *com.reg* function from the “quantreg” package [85]. All analyses were performed in R, version 4.4.1 [86].

## 5. Conclusions

The results of this study demonstrate that *P. tinctorum* and *U. barbata* possess efficient photoprotective mechanisms, including energy dissipation as heat, which enable their survival in high-light environments such as the dry landscapes of the Brazilian Savanna. In particular, the challenging physiognomies of Cerrado and Cerrado Ralo, where high DI_0_/RC levels led to reduced photochemical performance, were highlighted. However, *U. barbata* exhibited greater resilience to light stress than *P. tinctorum*, likely due to the presence of antioxidant metabolites such as usnic acid. Additionally, lichens sampled at higher stem positions and under elevated PAR levels dissipated less energy as heat and exhibited lower photochemical performance, indicating damage to PSII under these conditions. Conversely, when different spectral components were analyzed separately, increased light intensities reduced DI_0_/RC and enhanced Pi_ABS_, suggesting photodamage resistance in *P. tinctorum* and *U. barbata*. The ability of both species to adapt to high-light conditions, coupled with their physiological plasticity, supports their widespread distribution in these tropical ecosystems. As a future perspective, we propose conducting molecular and metabolomic studies to characterize the antioxidant compounds and identify the regulatory pathways involved in the photoprotection mechanisms of *U. barbata* and *P. tinctorum*. Additionally, we recommend investigating these species across different seasons and microhabitats to better understand their physiological plasticity and adaptive limits in response to varying light intensities.

## Figures and Tables

**Table 1:** Models used to test the effect of the number of landscape types, species, lichen height on the tree trunk, PAR, PPFD, PFDUV, PFDG, PFDR, PFDFR, and IRR on different chlorophyll *a* fluorescence parameters (ABS/RC, TR_0_/RC, ET_0_/RC, DI_0_/RC, PHI_DO_, PI_ABS_, PHI_PO_, PSI_0_, and PHI_E0_).

ABS/RC					
**Model**	**∆AICc**	**wAIC**	**K**	**AIC**	**Pr (>F)**
**Landscape + Species**	**0.0**	**0.6619**	**7**	**651.47**	**-**
**Landscape + Species + Height**	**1.4**	**0.3367**	**8**	**652.86**	**0.4332**
Landscape + Species + Height + PAR	12.6	0.0012	9	653.47	0.3686
Landscape + Species + Height + PFDUV + PFDG + PFDR + PFDFR + IRR + PAR	20.1	<0.001	14	648.87	0.0201 *
Landscape + Species + Height + PFDG + PFDR + PFDFR + IRR + PAR	20.3	<0.001	13	647.27	0.0127 *
Landscape + Species + Height + PFDFR + IRR + PAR	21.0	<0.001	11	651.28	0.0846
Landscape + Species + Height + + IRR + PAR	21.1	<0.001	10	655.07	0.4936
Landscape + Species + Height + PFDR + PFDFR + IRR + PAR	21.1	<0.001	12	648.67	0.0252 *
Landscape + Species + Height + PPFD + PFDUV + PFDG + PFDR + PFDFR + IRR + PAR	26.3	<0.001	15	648.98	0.0178 *
**TR_0_/RC**					
**Model**	**∆AICc**	**wAIC**	**K**	**AIC**	**Pr (>F)**
**Landscape + Species**	**0.0**	**0.74**	**7**	**169.25**	**-**
**Landscape + Species + Height**	**2.0**	**0.27**	**8**	**171.25**	**0.9820**
Landscape + Species + Height + PAR	18.1	<0.001	9	171.92	0.4151
Landscape + Species + Height + + IRR + PAR	31.2	<0.001	10	173.27	0.5754
Landscape + Species + Height + PFDFR + IRR + PAR	40.6	<0.001	11	174.57	0.6126
Landscape + Species + Height + PFDR + PFDFR + IRR + PAR	47.3	<0.001	12	174.03	0.3897
Landscape + Species + Height + PFDG + PFDR + PFDFR + IRR + PAR	52.6	<0.001	15	174.11	0.3074
Landscape + Species + Height + PFDUV + PFDG + PFDR + PFDFR + IRR + PAR	56.0	<0.001	13	174.29	0.2556
Landscape + Species + Height + PPFD + PFDUV + PFDG + PFDR + PFDFR + IRR + PAR	64.2	<0.001	14	171.47	0.0876
**ET_0_/RC**					
**Model**	**∆AICc**	**wAIC**	**K**	**AIC**	**Pr (>F)**
**Landscape + Species**	**0.0**	**0.75**	**7**	**81.903**	**-**
**Landscape + Species + Height**	**2.0**	**0.29**	**8**	**83.851**	**0.8206**
Landscape + Species + Height + PAR	16.3	<0.001	9	81.206	0.0955
Landscape + Species + Height + + IRR + PAR	30.2	<0.001	10	82.516	0.1456
Landscape + Species + Height + PFDFR + IRR + PAR	35.7	<0.001	11	78.455	0.0221 *
Landscape + Species + Height + PFDR + PFDFR + IRR + PAR	42	<0.001	12	76.390	0.0083 **
Landscape + Species + Height + PFDG + PFDR + PFDFR + IRR + PAR	48.5	<0.001	15	76.807	0.0089 **
Landscape + Species + Height + PFDUV + PFDG + PFDR + PFDFR + IRR + PAR	53.9	<0.001	13	78.213	0.0134 *
Landscape + Species + Height + PPFD + PFDUV + PFDG + PFDR + PFDFR + IRR + PAR	63.7	<0.001	14	75.926	0.0049 **
**DI_0_/RC**					
**Model**	**∆AICc**	**wAIC**	**K**	**AIC**	**Pr (>F)**
**Landscape + Species**	**0.0**	**0.6619**	**7**	**639.23**	**-**
**Landscape + Species + Height**	**1.3**	**0.3367**	**8**	**640.56**	**0.4131**
Landscape + Species + Height + PAR	12.6	0.0012	9	641.30	0.3802
Landscape + Species + Height + PFDUV + PFDG + PFDR + PFDFR + IRR + PAR	20.1	<0.001	14	636.63	0.0201 *
Landscape + Species + Height + PFDG + PFDR + PFDFR + IRR + PAR	20.3	<0.001	13	634.92	0.0121 *
Landscape + Species + Height + PFDFR + IRR + PAR	21	<0.001	11	638.79	0.0765
Landscape + Species + Height + + IRR + PAR	21.1	<0.001	10	642.94	0.5147
Landscape + Species + Height + PFDR + PFDFR + IRR + PAR	21.1	<0.001	12	636.22	0.0232 *
Landscape + Species + Height + PPFD + PFDUV + PFDG + PFDR + PFDFR + IRR + PAR	26.3	<0.001	15	637.08	0.0200 *
**PHI_DO_**					
**Model**	**∆AICc**	**wAIC**	**K**	**AIC**	**Pr (>F)**
**Landscape + Species**	**0.0**	**0.71**	**7**	**74.815**	**-**
**Landscape + Species + Height**	**1.8**	**0.29**	**8**	**75.516**	**0.6780**
Landscape + Species + Height + PAR	20.5	<0.001	9	74.815	0.4793
Landscape + Species + Height + + IRR + PAR	36.4	<0.001	10	73.076	0.6297
Landscape + Species + Height + PFDFR + IRR + PAR	45.2	<0.001	11	75.174	0.2122
Landscape + Species + Height + PFDR + PFDFR + IRR + PAR	52.6	<0.001	12	77.874	0.0615
Landscape + Species + Height + PFDG + PFDR + PFDFR + IRR + PAR	57.9	<0.001	13	80.515	0.0189 *
Landscape + Species + Height + PFDUV + PFDG + PFDR + PFDFR + IRR + PAR	65.3	<0.001	14	78.653	0.0322 *
Landscape + Species + Height + PPFD + PFDUV + PFDG + PFDR + PFDFR + IRR + PAR	77.4	<0.001	15	80.007	0.0167 *
**PI_ABS_**					
**Model**	**∆AICc**	**wAIC**	**K**	**AIC**	**Pr (>F)**
**Landscape + Species**	**0.0**	**0.76**	**7**	**140.86**	**-**
**Landscape + Species + Height**	**1.8**	**0.28**	**8**	**142.87**	**0.9989**
Landscape + Species + Height + PAR	19.2	<0.001	9	144.38	0.7888
Landscape + Species + Height + + IRR + PAR	32.9	<0.001	10	146.12	0.8639
Landscape + Species + Height + PFDFR + IRR + PAR	41.2	<0.001	11	145.94	0.5712
Landscape + Species + Height + PFDR + PFDFR + IRR + PAR	49.8	<0.001	12	147.12	0.5872
Landscape + Species + Height + PFDG + PFDR + PFDFR + IRR + PAR	52.1	<0.001	15	143.56	0.1576
Landscape + Species + Height + PFDUV + PFDG + PFDR + PFDFR + IRR + PAR	56.4	<0.001	13	144.51	0.1698
Landscape + Species + Height + PPFD + PFDUV + PFDG + PFDR + PFDFR + IRR + PAR	64.1	<0.001	14	140.69	0.0491 *
**PHI_PO_**					
**Model**	**∆AICc**	**wAIC**	**K**	**AIC**	**Pr (>F)**
**Landscape + Species**	**0.0**	**0.71**	**7**	**76.859**	**-**
**Landscape + Species + Height**	**1.8**	**0.29**	**8**	**75.026**	**0.6831**
Landscape + Species + Height + PAR	20.6	<0.001	9	74.305	0.4852
Landscape + Species + Height + + IRR + PAR	36.5	<0.001	10	72.591	0.6298
Landscape + Species + Height + PFDFR + IRR + PAR	45.2	<0.001	11	74.700	0.2113
Landscape + Species + Height + PFDR + PFDFR + IRR + PAR	52.5	<0.001	12	77.460	0.0598
Landscape + Species + Height + PFDG + PFDR + PFDFR + IRR + PAR	57.7	<0.001	13	80.246	0.0174 *
Landscape + Species + Height + PFDUV + PFDG + PFDR + PFDFR + IRR + PAR	65.1	<0.001	14	78.392	0.0297 *
Landscape + Species + Height + PPFD + PFDUV + PFDG + PFDR + PFDFR + IRR + PAR	77.1	<0.001	15	79.842	0.0149 *
**PSI_0_**					
**Model**	**∆AICc**	**wAIC**	**K**	**AIC**	**Pr (>F)**
**Landscape + Species**	**0.0**	**0.73**	**7**	**90.765**	**-**
**Landscape + Species + Height**	**2.0**	**0.27**	**8**	**88.765**	**0.9987**
Landscape + Species + Height + PAR	18.8	<0.001	9	90.286	0.1720
Landscape + Species + Height + + IRR + PAR	34.1	<0.001	10	89.339	0.2057
Landscape + Species + Height + PFDFR + IRR + PAR	43.2	<0.001	11	91.304	0.0737
Landscape + Species + Height + PFDR + PFDFR + IRR + PAR	51.0	<0.001	12	93.565	0.0253 *
Landscape + Species + Height + PFDG + PFDR + PFDFR + IRR + PAR	58.8	<0.001	13	93.652	0.0211 *
Landscape + Species + Height + PFDUV + PFDG + PFDR + PFDFR + IRR + PAR	65.2	<0.001	14	93.020	0.0228 *
Landscape + Species + Height + PPFD + PFDUV + PFDG + PFDR + PFDFR + IRR + PAR	75.0	<0.001	15	97.146	0.0042 **
**PHI_E0_**					
**Model**	**∆AICc**	**wAIC**	**K**	**AIC**	**Pr (>F)**
**Landscape + Species**	**0.0**	**0.74**	**7**	**143.35**	**-**
**Landscape + Species + Height**	**1.6**	**0.27**	**8**	**141.35**	**0.9899**
Landscape + Species + Height + PAR	20.3	<0.001	9	142.0	0.2610
Landscape + Species + Height + + IRR + PAR	36.6	<0.001	10	140.59	0.3541
Landscape + Species + Height + PFDFR + IRR + PAR	46.3	<0.001	11	142.54	0.1257
Landscape + Species + Height + PFDR + PFDFR + IRR + PAR	55.1	<0.001	12	144.26	0.0530
Landscape + Species + Height + PFDG + PFDR + PFDFR + IRR + PAR	62.7	<0.001	13	145.13	0.0321 *
Landscape + Species + Height + PFDUV + PFDG + PFDR + PFDFR + IRR + PAR	70.1	<0.001	14	143.97	0.0411 *
Landscape + Species + Height + PPFD + PFDUV + PFDG + PFDR + PFDFR + IRR + PAR	80.8	<0.001	15	147.75	0.0089 **

The models with Δ AICc < 2.0 are in bold type. AIC = Akaike value; AICc = AIC corrected by sample size and number of parameters in the model; wAIC = Akaike weight; K = number of parameters. Pr is the probability of the model in relation to the simplest model (Landscape + Species), obtained by the F-test. * = significant effect at 0.05 and ** = significant effect at 0.01.

## Data Availability

All the data relevant to this manuscript are available on request from the corresponding author.
